# The zoocenosis of the Aral Sea: six decades of fast-paced change

**DOI:** 10.1007/s11356-018-3807-z

**Published:** 2018-11-27

**Authors:** Nikolay Vasilevich Aladin, Valentina Ivanovna Gontar, Ljubov Vasilevna Zhakova, Igor Svetozarovich Plotnikov, Alexey Olegovich Smurov, Piotr Rzymski, Piotr Klimaszyk

**Affiliations:** 1 Zoological Institute of Russian Academy of Sciences, Saint-Petersburg, Russia; 2 Department of Environmental Medicine, Poznan University of Medical Sciences, Poznań, Poland; 3 Department of Water Protection, Adam Mickiewicz University, Poznań, Poland

**Keywords:** Aral Sea, Ecological disaster, Ichthyofauna, Aquatic macroinvertebrates, Aquatic restoration

## Abstract

During the last six decades, the water level of the Aral Sea, once one of the largest lakes in the world, has experienced a major human-driven regression followed by significant changes in salinity. These fast-paced alterations were initiated by the diversion of two rivers—the Amu Darya and Syr Darya—key players in the regulation of the water balance of the Aral Sea. Consequently, biological modifications took place leading to severe changes of the zoocenosis. This paper reviews the changes that have affected communities of fish and aquatic invertebrates in the Aral Sea since the 1950s. The reported alterations in biodiversity not only represent a natural response to a decrease in water level and a subsequent increase in salinity but also effects of non-native species introduction. The future prospects for invertebrates and fish in the Aral Sea, assuming that initiated restoration work is continued, are also discussed in this paper.

## Introduction

**Fig. 1:**
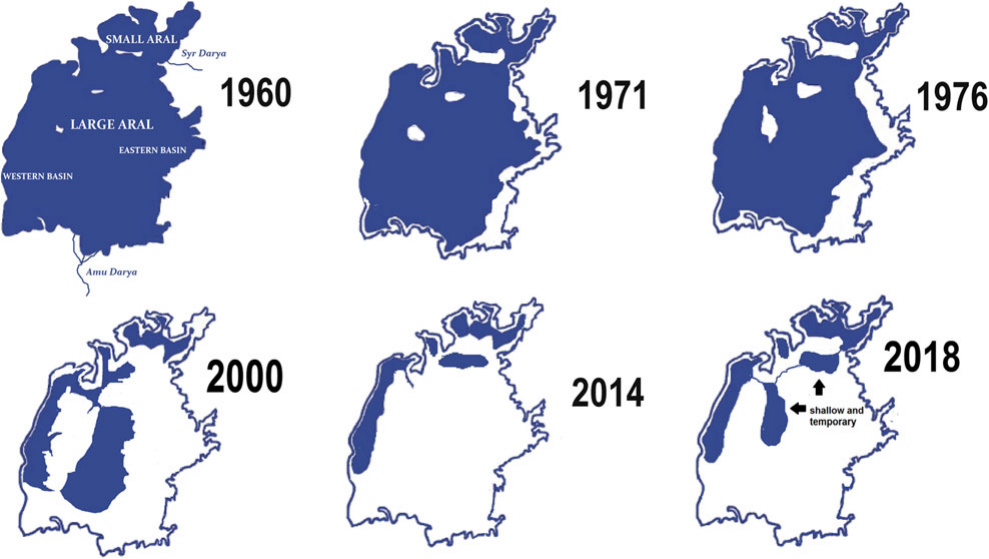
The profile of the Aral Sea during the last six decades (borders modified from Micklin (2016))

**Fig. 2:**
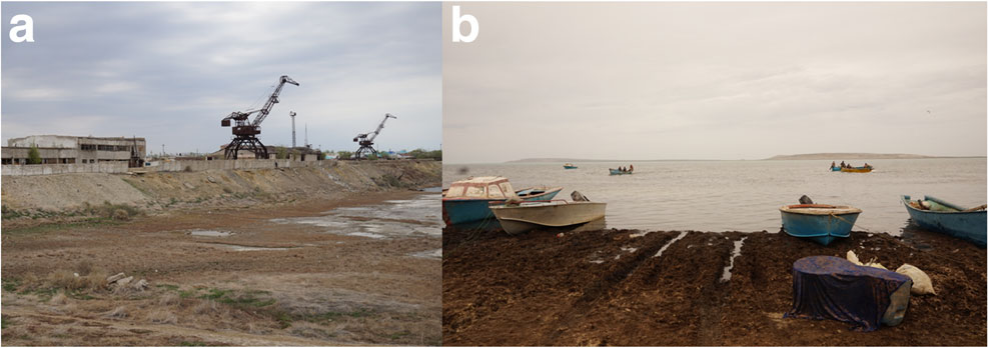
A once prosperous port of the Aral Sea in the city of Aralsk in Kazakhstan (**a**) and current areas of fishery on the Small Aral near Tastubek in Kazachstan (**b**). Photographs by authors

In the last six decades, the Aral Sea has suffered from an unprecedented human-driven, ecological disaster leading to rapid, wide-scale changes in its water level followed by a rise in salinity and modifications in biodiversity (Aladin and Potts 1992; Micklin 2014a, b; Singh et al. 2018). In the late 1950s, the second largest saline continental water body and fourth largest lake in the world, entered upon an inevitable decline as a result of a governmental decision to expand cotton agriculture, particularly in the territory of present day Uzbekistan. To create a sufficient water resource for such a purpose, the Syr Darya and Amu Darya rivers were diverted using canals. Because precipitation in the Aral Sea basin is very low and largely exceeded by evaporation, both rivers, the only surface water inflows to the Aral Sea, had always played a key role in the water balance of this reservoir. The great loss of inflowing water caused a massive change in the water level, changes in water chemistry (particularly in salinity), and diversification of the Aral into a few smaller water bodies (Fig. 1). A systematic regression of water level has led to the emergence of three separate basins: the Small Aral, the Eastern Large Aral, and the Western Large Aral. The cities of Aralsk (Kazakhstan) and Moynaq (Uzbekistan), once prosperous centers of fishery, have been largely distanced from the lake’s shoreline (Fig. 2). The loss of such an enormous water body has caused climate changes, economic crisis, and health issues over the entire region (Khan et al. 2004; Bosch et al. 2007; Aladin et al. 2009; Lioubimtseva 2014).

However, over the years, some actions to partially restore Aral Sea have been undertaken, dating back to 1990s when the first attempts to build a dam (known as the Dike Kokaral) to preserve the Syr Darya waters were made. Built using local sand and with largely limited funding, its first two constructions were washed away soon after the water level in the northern part of the Aral began to rise, subsequently increasing the pressure exerted on the dam. In 2005, funds from the World Bank enabled a proper dam to be constructed, and since then, the Syr Darya freshwaters have progressively contributed to the water level of the northern part of the Aral Sea, causing a simultaneous decrease in salinity (Aladin et al. 2009; Micklin 2014a, b). Periodically, and particularly in the spring seasons, an excess of water in the Northern Aral leads to a flow into the southern area through a sluice in the dike, although this is insufficient to overcome evaporation. Water contributions of the Amu Darya River have never been fully restored, thus the southern part of what was once a massive water body has now mostly vanished.

The rapid changes induced by human activity in the late 1950s were inevitably reflected in the biology of the Aral Sea, including zoocenosis and its diversity. Over the decades, a number of studies on the composition of fish and macroinvertebrates inhabiting the Aral waters have been conducted. Considering that future restoration works may be launched to expand the surface of the northern part of the Aral Sea, and hopefully also its southern basin, it is imperative not only to continue faunistic research but also to review the data which is currently available. Therefore, this paper presents changes in the zoocenosis (particularly macrozoobenthos, zooplankton, and fish) of the Aral Sea that have occurred since the late 1950s and further discusses its future prospects.

## Changes in water level and chemistry of the Aral Sea

During the last two millennia, the history of the Aral Sea has been a story of a dynamic change, of drops in water level, and transgressions. As the Aral Sea is a closed terminal lake, its water level and salinity depend on the ratio of river runoff, precipitation, and evaporation. According to the sedimentary and faunal record, the modern water drop, initiated in the 1950s, was preceded by two other deep regressions that occurred 2.1–1.3 ka cal BP with the water level falling to approximately as low as 10 m a.s.l., and 1.1–0.35 ka cal BP with a drop to 29 m a.s.l. These events were followed by intermediate transgressions, with the highest water level reaching as much as 54 m a.s.l. (Sorrel et al. 2006; Krivonogov et al. 2014). Nevertheless, for ~ 200 years prior to the beginning of the modern regression in the 1960s, the state of the Aral Sea remained quasi-stable with the water level fluctuating within a range of + 50 to + 53 m a.s.l. (Bortnik and Chistyayeva 1990). Before the modern regression, the Aral Sea was brackish with an average water salinity of 10.3‰ (Bortnik and Chistyayeva 1990).

Changes in water level were always reflected by an increase or decrease in salinity (Fig. 4), although one should note that salt composition in the Aral Sea reveals significant differences from that observed for oceans. The main difference includes the largely increased ratio of divalent ions (bicarbonate, calcium, magnesium, and sulfate) to monovalent ions (sodium and chlorine). This is due to the salt composition of the slightly mineralized (up to ~ 1–1.5‰) waters of the rivers flowing into the Aral in which the proportion of divalent ions is also higher (Bortnik and Chistyayeva 1990).

The peculiarities of the salinity regime of individual areas are related to their geographical location, morphometric features, and distance from the mouths of rivers, water circulation, and the intensity of water exchange. Due to the influence of the freshwaters, the mouths of the Amu Darya and Syr Darya were characterized by low salinity. However, salinity has always increased in the shoals and gulfs of the eastern coast and in the water area of the Karabayli archipelago. As a result of intensive evaporation under conditions of desert climate and difficult water exchange with the open sea, these areas were characterized by salinity reaching as much as 47–50‰ in the summer season (Bortnik and Chistyayeva 1990; Dengina 1959; Husainova 1960).

Increasing withdrawals of the Amu Darya and Syr Darya waters for the irrigation of cotton fields began in the 1960s and initiated the modern regression of the Aral Sea as evaporation began to exceed the inflow of freshwater. Since 1961, there has been a general increase in water salinity throughout the lake. During the first decade, salinity growth was slow, increasing only up to 11.5‰, while the water level fell from + 53 m a.s.l. in 1960 to + 51 m a.s.l. In 1974, the flow of the Syr Darya waters ceased because of the damming of the riverbed in its lower reaches. In 1982, the discharge of the Amu Darya waters into the sea via its main bed was terminated. Water inflow was completely absent in 1982, 1983, and 1985. Thus, after 1970, the decline in the water level of the Aral Sea systematically accelerated, reaching + 46 m a.s.l. by 1980 with a mean salinity of 17‰. In the 1980s, further reduction of the riverine water inflow led to the loss of the freshened zones that existed in front of the Amu Darya and Syr Darya deltas (Bortnik and Chistyayeva 1990).

The increase in the salinity of the Aral Sea waters led to further changes in its ion-salt composition. The proportion of divalent ions decreased due to the sequential precipitation of calcium carbonate, magnesium carbonate, gypsum, mirabilite, and halite (Bortnik and Chistyayeva 1990; Zavialov et al. 2012).

By 1988–1989, the water level had fallen to + 40 m a.s.l. while mean salinity had increased up to 30‰. The surface area was reduced by 40% with the water volume constituting only 33% of that observed in 1960. The coastline receded most substantially in the east, the southeast and south with large shallow bays completely vanished. The straits that connected the Small (Northern) and Large (Southern) Aral dried up, and the Aral Sea began to turn into a complex of residual water bodies (Aladin and Plotnikov 2008).

The fall in the level of the Small Aral ceased in 1988, when the total of inflowing water balanced the evaporation from its surface. However, desiccation of the Large Aral continued. In the spring of 1990, during the seasonal increase in the Syr Darya flow, the level of the Small Aral rose and water flowed from it to the Large Aral over a natural barrier—the dried-up Berg Strait. In 1992, this flow was blocked by an earthen dam (Aladin et al. 1995). However, this dam was repeatedly destroyed by increasing pressure, and following its collapse in 1999, it was no longer restored. In 2004–2005, a new dam (the Kokaral dam) was constructed, and has successfully supported the level the Small Aral at + 42 m a.s.l. (Aladin and Plotnikov 2008) and has led to a decrease of salinity. The Small Aral has gradually become a brackish water body. Currently, its mean salinity is lower than it was in the 1960s. In April–May of 2013, its mean salinity reached 5.3‰, with the highest salinity of 9.9‰ measured in Butakov Bay, whereas in the estuary zone of the Syr Darya, the salinity was at a level as low as 1.2–2.0‰ (Plotnikov et al. 2016).

Desiccation of the Large Aral and an increase in its salinity continued after its separation from the Small Sea (Fig. 1). By the end of the 1990s, the Large Aral had become hyperhaline. In 2000, salinity exceeded 60‰, and by 2004, it had reached 100‰. In autumn, 2009, the Large Sea was divided into three residual water bodies—the Western and Eastern basins, and the former Tschebas Bay. Salinity of the deep-water Western basin exceeded 100‰. The Eastern basin became a shallow water body and its salinity could now exceed as much as 200‰ (Aladin and Plotnikov 2008; Zavialov et al. 2012).

## Changes in zoocenosis

The biodiversity of the Aral Sea has always been low. The native fauna of free-living invertebrates being represented by less than 250 species with the domination (~ 80%) of those originating from continental freshwater, brackish, and saline water bodies. Representatives of Ponto-Caspian and marine Mediterranean-Atlantic fauna were also present. The largest number of species (> 50) was represented by groups of rotifers (Rotatoria) and crustaceans (Crustacea) (Mordukhai-Boltovskoi 1974; Plotnikov et al. 2016).

Compared to the largest continental saline water body, the Caspian Sea, several taxa of free-living invertebrates were absent in the Aral Sea. No sponges (Porifera) or polychaete worms (Polychaeta) were found there. Higher crustaceans (Malacostraca) were represented by only one species of Amphipoda while Mysida, Cumacea, Isopoda, and Decapoda were not present. Among cladocerans (Cladocera) belonging to the Onychopoda order, only one species from the *Cercopagis* genus was present with no species of the *Apagis*, *Cornigerius*, and *Caspievadne* genera. There were no copepod (Copepoda) species from families Centropagidae and Temoridae of the order Calanoida.

The native zooplankton (excluding protozoans) was represented by rotifers, cladocerans (17 species), and copepods (16 species). The most common cladocerans included *Ceriodaphnia reticulata*, *Coronatella rectangula*, *Diaphanosoma brachyurum*, *Ceriodaphnia cornuta*, *C. pulchella*, *Daphnia longispina*, *Moina micrura*, *Chydorus sphaericus*, *Bosmina longirostris*, and *Polyphemus pediculus*—freshwater; *Cercopagis pengoi aralensis*, *Evadne anonyx*, *Podonevadne camptonyx*, and *P. angusta*—Ponto-Caspian endemics; *Moina mongolica*—a widely euryhaline halophile inhabiting continental saline waters. The most common copepods were *Arctodiaptomus salinus* that inhabits continental saline waters, freshwater *Mesocyclops leuckarti*, marine *Halicyclops rotundipes aralensis*, and the widely euryhaline *Megacyclops viridis* (Mordukhai-Boltovskoi 1974; Plotnikov et al. 2016).

The native benthic fauna (excluding Protozoa) were represented by nematodes (at least 10 species), turbellarians (12 species; among which *Kirgisella forcipata* and *Gieysztoria bergi* are considered to be endemic), bryozoans (3 species), oligochaetes (10 species, predominated by *Psammoryctides albicola*), ostracods (11 species, *Cyprideis torosa* being the most numerous), harpacticoids (15 species; among which *Schizopera aralensis*, *S. reducta*, and *Enhydrosoma birsteini* are considered to be endemic), larvae of insects (27 species with domination of *Chironomus behningii*), bivalves *Dreissena polymorpha aralensis*, *D. p. obtusecarinata* and *D. caspia pallasi*, *Hypanis vitrea bergi*, *H. minima minima*, *H. m. sidorovi*, *Cerastoderma rhomboides rhomboides*, and *C. isthmicum*, gastropods *Theodoxus pallasi* and *Caspiohydrobia* spp., and amphipod *Dikerogammarus aralensis*. Brackish water mollusks *Dreissena* spp., *Hypanis* spp. and *T. pallasi* and halophiles *Caspiohydrobia* spp. predominated (Mordukhai-Boltovskoi 1974; Plotnikov et al. 2016). Aboriginal malacofauna was poorly represented with only five species of bivalve mollusks (Bivalvia) of the Cardiidae family. Among gastropods (Gastropoda), no species of the genera *Pyrgula*, *Turricaspia*, *Caspia*, *Andrusovia*, *Pseudoamnicola*, and *Tenellia* were noted (Birstein et al. 1968; Mordukhai-Boltovskoi 1974).

Initially, the native ichthyofauna was also poorly represented by only 20 species: ship sturgeon *Acipenser nudiventris*, Aral trout *Salmo trutta aralensis*, pike *Esox lucius*, roach *Rutilus rutilus aralensis*, ide *Leuciscus idus oxianus*, asp *Aspius aspius iblioides*, rudd *Scardinius erythropthalmus*, Turkestan barbel *Barbus capito conocephalus* and Aral barbel *B. brachycephalus brachycephalus*, bream *Abramis brama orientalis*, white-eyed bream *A. sapa aralensis*, shemaya *Chalcalburnus chalcoides aralensis*, sabrefish *Pelecus cultratus*, crucian carp *Carassius carassius gibelio*, common carp *Cyprinus carpio aralensis*, catfish *Silurus glanis*, stickleback *Pungitius platygaster aralensis*, pike perch *Stizostedion lucioperca*, perch *Perca fluviatilis*, and ruff *Gymnocephalus cernuus*. In general, fish fauna was freshwater and euryhaline, and there were no fish species typical of a marine environment. Except for stickleback, all fish in the Aral Sea were semi-anadromous or anadromous (Ermakhanov et al. 2012).

In an attempt to increase the fish productivity of the Aral Sea, commercial fish species and selected invertebrates that serve as their feed were introduced in the second half of the twentieth century. However, hydropower construction in the 1950s–1960s and the further expansion of irrigated agricultural areas in the Amu Darya and Syr Darya basins inevitably led to a significant reduction in the flow of these rivers and an increase in the salinity of the Aral Sea. Therefore, introduction of salt-tolerant fish and invertebrates was required, although not all plans were successfully realized in this respect.

The planktophage Baltic herring (*Clupea harengus*) which was not recommended for acclimatization in the Aral Sea, was introduced in 1954–1956. This had significant consequences, particularly in the zooplankton community. Before its introduction, no obligatory planktophages were recorded. This species prefers large planktonic crustaceans and it reduced the density of *Arctodiaptomus salinus*, a species of low fertility and extended life cycle (only one generation/year) (Lukonina 1960; Yablonskaya and Lukonina 1962; Karpevich 1975; Kortunova 1975). Before the introduction of the Baltic herring, more than 70% of the zooplankton biomass consisted of only this crustacean. As a result of the introduction of Baltic herring, as well as atherine and gobies, the abundance and biomass of zooplankton sharply decreased. This particularly concerned the crustaceans *A. salinus*, *Cercopagis pengoi aralensis*, *Moina mongolica*, *Ceriodaphnia reticulata*, and cyclopids. The average summer biomass of zooplankton fell more than tenfold. This, in turn, led to the mass death of herring and atherine from starvation (Osmanov 1961; Kortunova and Lukonina 1970; Kortunova 1975). As a result, the numbers of plankton-eating fish in the Aral Sea was never able to reach a high level again.

In 1954–1956, during an unsuccessful attempt to introduce mullets (*Mugil* sp.) from the Caspian, the shrimp *Palaemon elegans* was also inadvertently introduced and quickly settled throughout the Aral Sea. This species is characterized by a wide range of tolerance to temperature and salinity, and is an invader currently known to induce adverse effects in native ecosystems (Janas et al. 2013). In the Aral Sea, *P. elegans* first caused a decrease in the number, and by 1973, the complete disappearance of the highly euryhaline *Dikerogammarus aralensis*, although this amphipod still remains in the Syr Darya and the lakes in its lower reaches (Mordukhai-Boltovskoi 1972; Aladin and Potts 1992). At the same time, some non-commercial fish were also introduced accidently: atherine *Atherina boyeri caspia*, pipefish *Syngnatus abaster caspius*, and gobies—Caucasian dwarf goby *Knipowitschia caucasica*, sand goby *Neogobius fluviatilis*, round goby *N. melanostomus*, syrman goby *N. syrman*, bighead goby *N. kessleri*, and tubenose goby *Proterorchinus marmoratus* (Karpevich 1975; Ermakhanov et al. 2012).

In 1958–1960, Ponto-Caspian mysids from the Don delta were introduced. These crustaceans can inhabit environments of salinity of up to 17–20‰. Of the three introduced species, *Paramysis lacustris*, *P. intermedia*, and *P. baeri*, only the first two were successfully naturalized. Another species of the *Paramysis* genus, *P. ullskyi*, underwent auto-acclimatization in the Aral from water reservoirs located in the course of the Syr Darya river (Karpevich 1975).

In the early 1960s, some marine euryhaline invertebrates—the polychaete worm *Hediste diversicolor* and bivalve mollusk *Syndosmya segmentum*—were introduced successfully from the Sea of Azov as valuable and accessible food for benthophage fish (Karpevich 1975).

In 1965 and in the 1970s, the highly productive (6 generations per year) marine planktonic crustacean *Calanipeda aquaedulcis* was introduced from the Sea of Azov in order to restore and increase the productivity of zooplankton after the extermination of *Arctodiaptomus salinus* by Baltic herring and atherine. This copepod became one of the dominating species in the zooplankton of the Aral Sea and replaced the native copepod *Arctodiaptomus salinus* and the cladoceran *Moina mongolica*. The North American mud crab *Rhithropanopeus harrisii tridentatus* was accidentally introduced (as planktonic larvae) together with *C. aquaedulcis* (Mordukhai-Boltovskoi 1972; Karpevich 1975; Plotnikov et al. 2016).

In 1960–1961, five species of commercial fish were introduced in the Aral Sea of which three were introduced advisedly: macrophytophage grass carp *Ctenopharyngodon idella*, phytoplanktophage silver carp *Hypophtalmichthys molitrix*, and the filter feeder, consuming phytoplankton, zooplankton, and detritus, bighead carp *Aristichthys nobilis*. Two other species, benthophage black carp *Mylopharyngodon piceus* and the predator snakehead *Channa argus warpachowski*, were introduced accidentally during fish works. They are freshwater species that prefer those areas of the Aral characterized by lowered salinity (Karpevich 1975; Ermakhanov et al. 2012).

**Fig. 3:**
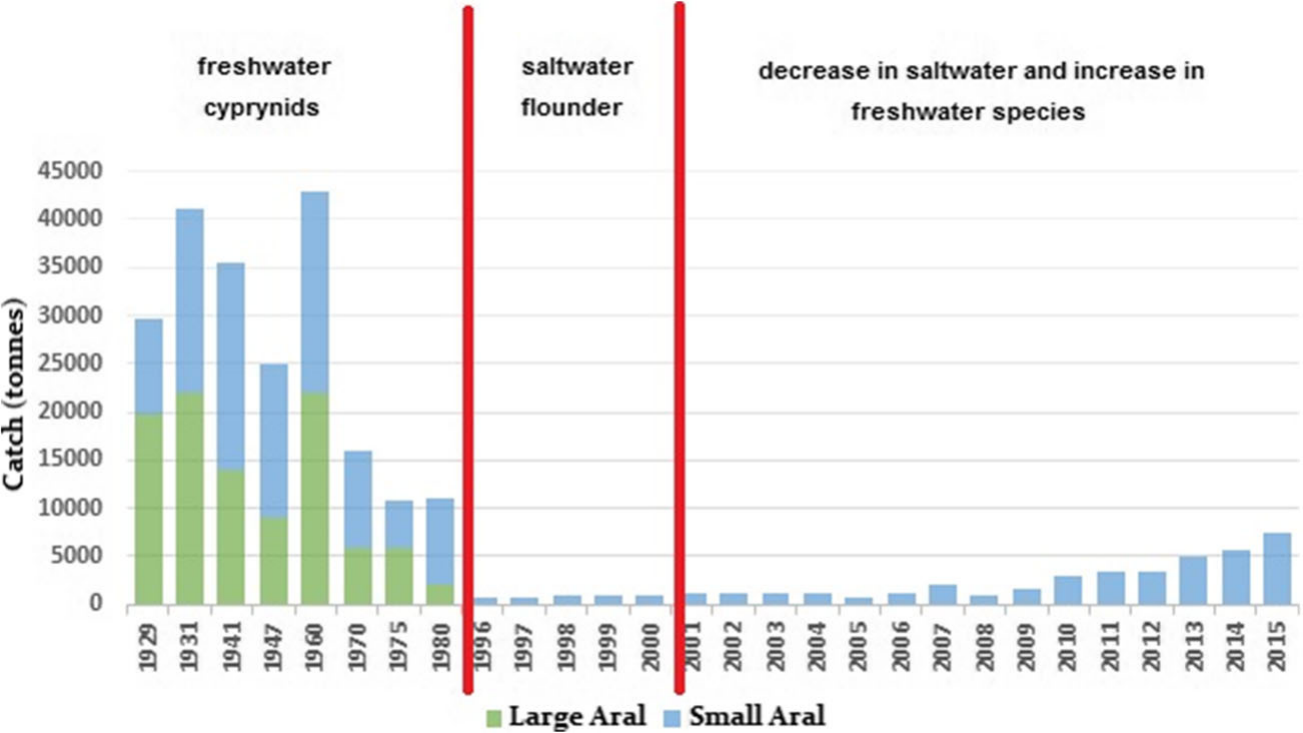
The commercial fish catch and fish structure under changing water level and salinity in the Aral Sea (based on Aladin et al. 2017)

**Fig. 4:**
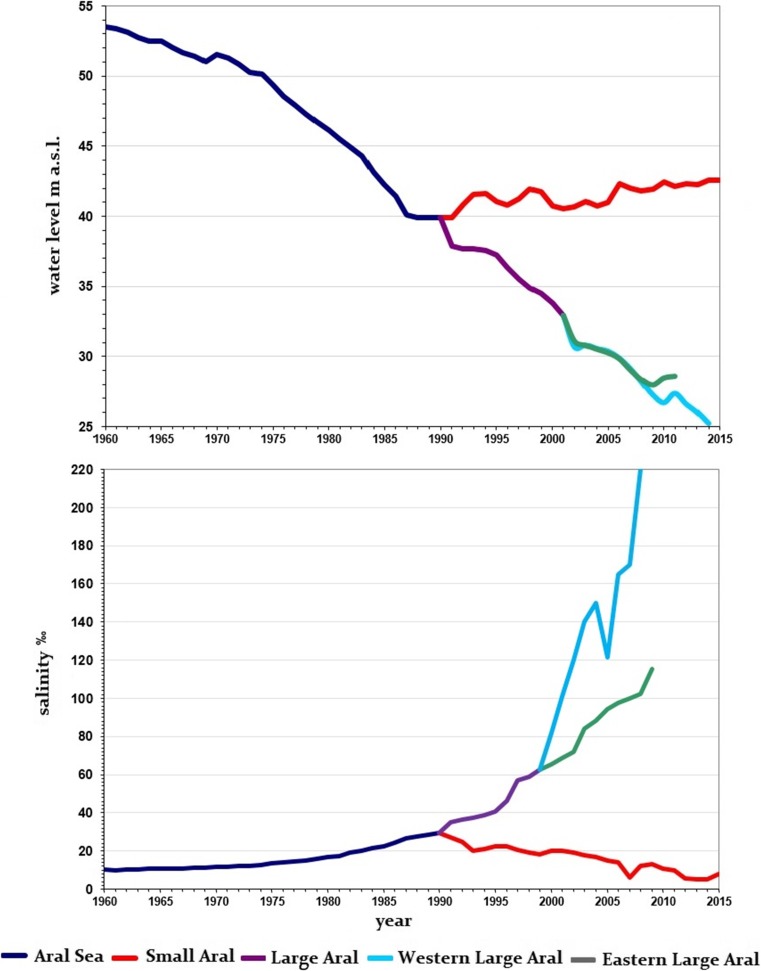
Changes in water level and salinity of the Aral Sea over the last six decades (based on Plotnikov (2013))

From the 1960s, the changing water salinity became the main factor affecting the Aral Sea biota, including its fish community (Table 1). The modification of ichtyofauna was also reflected by a decrease in commercial fishing (Fig. 3), having a profoundly adverse effect on the regional economy (Karimov et al. 2005). During the period of 1961–1970, Aral Sea desiccation and salinity increase occurred very slowly. Salinity increased by only 1.5‰, and by 1971, it had reached 11.5‰ (Fig. 4). At this early stage of the Aral Sea’s modern regression, changes in the biota were mostly the result of the introduction of new fish and invertebrate species. It is possible that *Hediste diversicolor*, along with increasing water salinity, had been responsible for a diminution in the numbers of chironomids and oligochaetes in the Aral Sea (Karpevich 1975).

**Table 1:** Species composition of the Aral Sea ichthyofauna (prepared from data provided by Ermakhanov et al. 2012)

Species	Years				Status
	1950	1960–1979	1980–1990	1991–2004	
Acipenseridae					
Ship sturgeon *Acipenser nudiventris*	+	+	–	–	C, E
Salmonidae					
Aral trout *Salmo trutta aralensis*	+	+	–	–	C, E
Clupeidae					
Baltic herring *Clupea harengus membras*	–	+	+	+	I
Esocidae					
Pike *Esox lucius*	+	+	–	+	C
Cyprinidae					
Aral roach *Rutilus rutilus aralensis*	+	+	–	+	C
Orfe *Leuciscus idus oxianus*	+	+	–	+	C
Asp, zherekh *Aspius aspius iblioides*	+	+	–	+	C
Rudd *Scardinius erythropthalmus*	+	+	–	+	C
Turkestan barbel *Barbus capito conocephalus*	+	+	–	–	C, R
Aral barbel *Barbus brachycephalus brachycephalus*	+	+	–	+	C, R
Bream *Abramis brama orientalis*	+	+	–	+	C
White-eye bream *Abramis sapa aralensis*	+	+	–	+	C
Aral shemaya *Chalcalburnus chalcoides aralensis*	+	+	–	+	C
Sabrefish *Pelecus cultratus*	+	+	–	+	C
Crucian carp *Carassius carassius gibelio*	+	+	–	+	C
Carp *Cyprinus carpio aralensis*	+	+	–	+	C
Grass carp *Ctenopharyngodon idella*	–	+	–	+	I, C
Silver carp *Hypophtalmichthys molitrix*	–	+	–	+	I, C
Spotted silver carp *Aristichtys nobilis*	–	+	–	+	I, C
Black carp *Mylopharyngodon piceus*	–	+	–	+	I, C
Syngnathidae					
Black-striped pipefish *Syngnathus abaster caspius*	–	+	?	?	I
Atherinidae					
Caspian atherine *Atherina boyeri caspia*	–	+	+	+	I
Gobiidae					
Caucasian dwarf goby *Knipowitschia caucasica*	–	+	+	+	I
Sand goby *Neogobius fluviatilis*	–	+	+	+	I
Round goby *Neogobius melanostomus*	–	+	+	+	I
Syrman goby *Neogobius syrman*	–	+	+	+	I
Tubenose goby *Proterorchinus marmoratus*	–	+	+	+	I
Bighead goby *Neogobius kessleri*	–	+	+	+	I
Siluridae					
Wels *Silurus glanis*	+	+	–	+	C
Gasterostidae					
Nine-spined stickleback *Pungitius platygaster aralensis*	+	+	+	+	
Percidae					
Pike perch, zander *Stizostedion lucioperca*	+	+	–	+	C
Perch *Perca fluviatilis*	+	+	–	+	C
Ruff *Gymnocephalus cernuus*	+	+	–	–	
Channidae					
Snakehead *Channa argus warpachowskii*	–	+	–	+	I, C
Pleuronectidae					
Flounder-gloss *Platichthys flesus*	–	–	+	+	I, C

+, present; –, absent; ?, no data; *I*, introduced; *C*, commercial; *R*, in Red Book; *E*, extinct

Nevertheless, the small increase in salinity led to a reduction in the total habitat for the bivalves *Dreissena* spp. and a significant decrease in their total number after 1964. By 1967, it had already decreased 40-fold. By the end of the 1960s, the number and area of the gastropod *Theodoxus pallasi* had also fallen, mostly due to a reduction in its preferred area, solid bottom, because of shoreline retreat (Plotnikov et al. 2014).

In 1971–1976, invertebrate fauna of the Aral Sea passed through the first crisis caused by salinization over the upper limit of 12–13 g/l of the first barrier of salinity. Exceeding this limit of water salinity became an obstacle for the further existence of species of freshwater origin. The most species-rich, freshwater component of fauna gradually disappeared. From the 21 species of rotifers that survived the crisis, only some species of *Synchaeta* were common and numerous. The freshwater cladocerans *Coronatella rectangula* and *Ceriodaphnia reticulata* disappeared, and by 1975, only Ponto-Caspian species remained. Only 16 species of Copepoda survived the first crisis. Instead of freshwater *Mesocyclops leuckarti*, the marine *Halicyclops rotundipes aralensis* became the most numerous. The least euryhaline species of Harpacticoida began to disappear (Plotnikov et al. 2014).

All subspecies of mollusks *Hypanis* disappeared completely and became extinct after 1977. *Cerastoderma rhomboides rhomboides* was also no longer found, and its ecological niche was taken by *C. isthmicum*. Salinity exceeding 12–14‰ favored euryhaline *Syndosmya segmentum*. From 1973, oligochaetes were no longer found, partially due to salinity changes but also the introduction of the polychaete worm *Hediste diversicolor*. By 1974 most of the larval chironomid species had disappeared and only *Chironomus salinarius* and *Ch. halophilus* remained. Owing to the increased salinity, the abundance of *Caspiohydrobia* spp. began to increase. Species diversity of ostracods decreased but *Cyprideis torosa* remained common. After 1977, mysids were absent from the Aral Sea surviving only in the rivers and their deltas. In the 1970s, *Dreissena polymorpha aralensis* disappeared from the Aral Sea, remaining exclusively in the Syr Darya and the lakes connected with it, while *D. p. obtusecarinata* and later *D. caspia pallasi* became extinct (Plotnikov et al. 2014; Plotnikov et al. 2016).

Despite the continuing rise in salinity, the first crisis period transitioned into a period of relative stability between 1977 and 1985. The most common species of Cladocera was *Pododevadne camptonyx*. The cladoceran *Cercopagis pengoi aralensis* survived in the least saline regions until 1980. The most abundant copepod was *Calanipeda aquaedulcis* (Plotnikov et al. 2014).

By 1987 salinity of the Aral Sea had risen to 27‰ (Fig. 4). Crossing the second salinity barrier (27–32‰) biota entered the period of the second crisis during which the next reduction of species diversity occurred. The remaining Ponto-Caspian cladocerans disappeared. Within the community of zooplankton, only some species of rotifers, *Calanipeda aquaedulcis*, *Halicyclops rotundipes aralensis*, and *Megacyclops viridis* remained. In the benthic fauna, only *Cerastoderma isthmicum*, *Caspiohydrobia* spp., *Cyprideis torosa*, *Hediste diversicolor*, *Syndosmya segmentum*, *Rhithropanopeus harrisii tridentata*, and *Palaemon elegans* and some harpacticoids survived. After the second crisis, there were only marine species, euryhaline species of marine origin, and representatives of euryhaline halophilic fauna of inland saline waters. *S. segmentum* had become the major component of the benthic fauna and replaced the extinct mollusks from the *Dreissena* and *Hypanis* genera (Plotnikov et al. 2014).

Fishing ceased in the early 1980s when freshwater fish disappeared from the sea; only stickleback, Baltic herring, atherine, and gobies survived as they can thrive in saline ecosystems. The Aral Sea lost its importance for the fishing industry causing economic consequences for the region (Figs. 2 and 3). In 1979–1987, Black Sea flounder-gloss *Platichthys flesus* was successfully introduced to revive the fisheries, and was fished commercially for some time (Ermakhanov et al. 2012).

A significant decrease in salinity and the formation of a highly freshened zone near the Syr Darya delta opened a possibility of a natural reintroduction of many freshwater and brackish water invertebrate species associated with the Syr Darya, its lower reaches and associated lakes, or invertebrate species with resting eggs that retain their viability for a long time (Plotnikov et al. 2014, 2016). The relative stabilization of the hydrological regime and freshening of the water of the Small Aral Sea promoted important commercial fish species including carp, bream, zander, and asp. However, fish such as ship sturgeon *Acipenser nudiventris* and the Aral trout *Salmo trutta aralensis* did not return to the Aral as their migration routes to spawning areas in the rivers had been blocked by dams (Ermakhanov et al. 2012). At the same time, the sharp decrease in salinity became unfavorable for the species of marine fauna and fauna of saline continental water bodies, for example, the density of benthic fauna mollusks *Cerastoderma isthmicum* and *Caspiohydrobia* spp., decreased significantly due to the lowered salinity (Plotnikov et al. 2016).

The decrease in salinity has led to the reappearance of freshwater rotifers such as *Filinia longiseta*, *Asplanchna priodonta*, and *Brachionus calyciflorus* in the Small Aral Sea. The biodiversity of planktonic crustaceans has also increased significantly due to the reappearance of *Bosmina longirostris*, *Chydorus sphaericus*, *Diaphanosoma brachyurum*, *Ceriodaphnia reticulata*, *Podonevadne angusta*, *Evadne anonyx*, and copepods *Phyllodiaptomus blanci*, *Cyclops vicinus*, *Mesocyclops leuckarti*, and *Acanthocyclops viridis*. The cladoceran *Moina mongolica* which became extinct in the 1970s has also reappeared. The marine copepod *Halicyclops rotundipes aralensis* has either currently decreased in number or completely disappeared. The most common planktonic invertebrate species in the Small Aral are now represented by the rotifers *Synchaeta* spp., *Keratella quadrata*, *Brachionus quadridentatus*, *B. plicatilis*, and the cladoceran *Evadne anonyx*; and copepods *Calanipeda aquaedulcis* and *Cyclops vicinus*. A mysid, *Paramysis intermedia*, has returned to the Small Aral from the lower reaches of the Syr Darya. Moreover, the bivalve mollusk *Dreissena polymorpha aralensis* has been successfully reintroduced in the freshened zones. At least eight species of larval chironomids were identified in the Small Aral during the most recent inventory performed in 2013 (Plotnikov et al. 2016).

The transformation of the Large Aral into a hyperhaline water body led to a further reduction of biodiversity. The majority of representatives of marine fauna became extinct and only those invertebrate species most resistant to high salinity, particularly nematodes, have survived. The rotifer *Synchaeta* spp. disappeared, but *Brachionus plicatilis* and *Hexarthra* sp. have become common. Copepods *Calanipeda aquaedulcis*, *Halicyclops rotundipes aralensis*, and *Megacyclops viridis* have disappeared. The halophilic cyclopid *Apocyclops dengizicus* was introduced naturally. The number of species of harpacticoides decreased, only the most halotolerant species remained, *Cletocamptus retrogressus*, and, plausibly, *C. confluens* and *Nitocra lacustris*. Among ostracods, *Cyprideis torosa* remained, and the euryhaline halophile *Eucypris mareotica* settled widely. All Malacostraca disappeared. Halophilic infusoriums *Frontonia marina* and *Fabrea salina* as well as larvae of the chironomid *Baeotendipes noctivaga* appeared. Some fish still survived, gobies, although Baltic herring, atherine, and flounder became extinct. All conditions for auto-introduction of the halobiont brine shrimp *Artemia* were formed, and this crustacean was identified in 1998. Here, *Artemia* is represented by parthenogenetic populations, usually united under the name *A. parthenogenetica* (Plotnikov et al. 2016).

As a result of the water discharge from the Small Aral through the Kokaral dam to the south, one more water body, the Central Aral (Micklin 2016), has appeared. It is a shallow, very unstable lake. Together with water from the Small Sea, a large number of valuable commercial fish are brought to the Central Aral. However, the salinity (~ 70‰) in the west of this lake is too high for their survival.

## Future prospects

Further perspectives for the biodiversity (including zoocenosis) of the residual water bodies of what was once the Aral Sea depend primarily on salinity changes. Its further decrease in the Small Aral may affect the biodiversity of invertebrates, and negatively affect marine species and species from the fauna of saline continental water bodies of the arid zone. Therefore, the survival of mollusks such as *Cerastoderma isthmicum*, *Syndosmya segmentum*, *Caspiohydrobia* spp., the polychaete worm *Hediste diversicolor*, and the shrimp *Palaemon elegans* will be threatened. If the shrimp disappears, a return of the amphipod *Dikerogammarus aralensis* will be possible. Rotifers *Synchaeta* spp. and cladocerans from the Podonidae family may also become extinct. Members of Foraminifera and Infusoria will disappear, as well as most of the turbellarians. It is difficult to forecast changes in nematode diversity due to a lack of consistent data on their presence. Very low salinity will allow all known species of ostracods, except *Limnocythere aralensis*, to exist in the Small Aral. Low salinity will be favorable for the larvae of freshwater chironomids. Significant freshening of waters will be unfavorable for marine and halophilic copepods. Only freshwater and widely euryhaline species will remain: among Calanoida—*Calanipeda aquaedulcis* and *Phyllodiaptomus blanci*. From Cyclopoida, *Halicyclops rotundipes aralensis* will disappear, but all other (freshwater) species are expected to remain. Among Harpacticoida, species that do not tolerate freshwaters will become extinct (Plotnikov et al. 2016).

Currently, a project has been proposed to construct a dam in the neck of Bolshoy Sarycheganak Bay with a spillway into the main water area of the Small Aral, and a channel from the Aklak control structure to divert part of the Syr Darya flow into this bay. If this work is realized, the Small Sea should become a cascade of two water bodies that differ in salinity. The bay areas will be almost freshwater (salinity < 2‰) and inhabited by hydrobionts introduced from the Syr Darya while marine and brackish organisms will disappear. In turn, the main part of the Small Sea will be brackish. There is also an alternative project to reconstruct and elevate the Kokaral dam. In this case, the level and area of the entire Small Aral will increase, and the entire Small Aral will remain brackish water with a freshened zone only in the vicinity of the Syr Darya delta (Plotnikov et al. 2016).

The forecast for biodiversity of the hyperhaline residual water bodies of the Large Aral is different as their water balance remains negative. In the absence of any water inflow from the Amu Darya, one cannot expect any rapid stabilization of level or salinity of these residual water bodies—any decrease in salinity is virtually impossible. With no action undertaken to impede salinity growth in the Western Large Aral and Tschebas Bay, the decline in their, already low, biodiversity will continue. It is likely that the ostracod *Cyprideis torosa* and rotifer *Brachionus plicatilis* will become extinct. Rotifers from the *Hexarthra* genus will also disappear. Harpacticoides *Cletocamptus confluens*, *Nitocra lacustris*, *C. retrogressus*, and the cyclopid *Apocyclops dengizicus* are more tolerant to salinization. In the future, disappearance of the ostracod *Eucypris inflata* and larvae of chironomid *Baeotendipes noctivaga* is possible. Halophilic infusoriums *Frontonia marina* and *Fabrea salina* will also become extinct. Under circumstances of continuous salinization, the fauna of free-living invertebrates of these residual water bodies of the Large Aral will be represented only by *Artemia* that can withstand salinity up to 350‰. If salinity exceeds the upper limit of the saline tolerant range of *Artemia*, these water bodies will turn into a likeness of the Dead Sea. In any case, the biodiversity changes will depend on the extent to which the salinity of the Western Large Aral and Tschebas Bay will increase (Plotnikov et al. 2016).

Fauna of the free-living invertebrates of the Eastern Large Aral Sea, before its desiccation, most likely represented only by *Artemia*, can be restored even after extinction, when this residual water body will once again receive water from the Amu Darya. The source for the restoration of the *Artemia* population will be constituted of cysts that will remain on its dried bottom and/or cysts which are carried by wind from other hyperhaline water bodies (Plotnikov et al. 2016).

## Conclusions

The water level and salinity of the Aral Sea has always been subject to dynamic changes as the survival of this water body depends only on two rivers. Their diversion in late 1950s for agricultural reasons caused a systematic loss of inflowing water, an increase in salinity, and rapid changes to fish and macroinvertebrates. The situation in the northern part of the Aral is currently stable due to the existence of the Kokaral dam. Further work is required to expand its surface and to restore the southern part which is now mostly vanished. It is beyond any doubt that the future of the Aral Sea and its biodiversity, which is also the future of fishery and related economics, depends now almost entirely on human decisions.
